# Adeno-Associated Viral Vector Serotype DJ-Mediated Overexpression of N171-82Q-Mutant Huntingtin in the Striatum of Juvenile Mice Is a New Model for Huntington’s Disease

**DOI:** 10.3389/fncel.2018.00157

**Published:** 2018-06-12

**Authors:** Minhee Jang, Seung Eun Lee, Ik-Hyun Cho

**Affiliations:** ^1^Department of Convergence Medical Science, College of Korean Medicine, Kyung Hee University, Seoul, South Korea; ^2^Virus Facility, Research Animal Resource Center, Korea Institute of Science and Technology (KIST), Seoul, South Korea; ^3^Brain Korea 21 Plus Program and Institute of Korean Medicine, College of Korean Medicine, Kyung Hee University, Seoul, South Korea

**Keywords:** Huntington’s disease, adeno-associated viral vector serotype DJ, mutant huntingtin, N171-82Q, polyglutamine expansion

## Abstract

Huntington’s disease (HD) is an autosomal-dominant inherited neurodegenerative disorder characterized by motor, psychiatric and cognitive symptoms. HD is caused by an expansion of CAG repeats in the huntingtin (*HTT*) gene in various areas of the brain including striatum. There are few suitable animal models to study the pathogenesis of HD and validate therapeutic strategies. Recombinant adeno-associated viral (AAV) vectors successfully transfer foreign genes to the brain of adult mammalians. In this article, we report a novel mouse model of HD generated by bilateral intrastriatal injection of AAV vector serotype DJ (AAV-DJ) containing N171-82Q mutant *HTT* (82Q) and N171-18Q wild type *HTT* (18Q; sham). The AAV-DJ-82Q model displayed motor dysfunctions in pole and rotarod tests beginning 4 weeks after viral infection in juvenile mice (8 weeks after birth). They showed behaviors reflecting neurodegeneration. They also showed increased apoptosis, robust glial activation and upregulated representative inflammatory cytokines (tumor necrosis factor-alpha (TNF-α) and interleukin (IL)-6), mediators (cyclooxygenase-2 and inducible nitric oxide synthase) and signaling pathways (nuclear factor kappa B and signal transducer and activator of transcription 3 (STAT3)) in the striatum at 10 weeks after viral infection (14 weeks after birth) via successful transfection of mutant *HTT* into neurons, microglia, and astrocytes in the striatum. However, little evidence of any of these events was found in mice infected with the AAV-DJ-18Q expressing construct. Intrastriatal injection of AAV-DJ-82Q might be useful as a novel *in vivo* model to investigate the biology of truncated N-terminal fragment (N171) in the striatum and to explore the efficacy of therapeutic strategies for HD.

## Introduction

Huntington’s disease (HD) is an inherited progressive neurodegenerative disorder characterized by involuntary abnormal movements (chorea), cognitive decline and emotional as well as psychiatric disturbances (Damiano et al., 2010; Jacobsen et al., 2011; Gil-Mohapel et al., 2014). HD typically becomes apparent at 35–45 years of age and progresses toward death within 15–20 years after the appearance of the first clinical symptoms (Ceccarelli et al., 2016). HD is caused by an abnormal expansion of a CAG codon ≥36 repeats located in exon one of the huntingtin gene (*HTT*) on chromosome 4 that confers a toxic function to the protein (Damiano et al., 2010; Jacobsen et al., 2011; Gil-Mohapel et al., 2014). The expansion encodes a prolonged polyglutamine sequence that results in conformational change of the *HTT* protein and induces the formation of intranuclear inclusions of mutant *HTT* in various areas of the brain. This mutation leads to neuronal loss and neuronal degeneration, most prominently in the striatum (Damiano et al., 2010; Jacobsen et al., 2011; Gil-Mohapel et al., 2014). On postmortem analysis, ubiquitinated intranuclear inclusions are observed, suggesting abnormal processing/folding of the polyglutamine domain in affected cells. However, the pathogenic mechanisms leading to neurodegeneration are unclear.

There is no established treatment to prevent or to delay the onset or forestall the progression of HD (Wyant et al., 2017). A critical aspect for drug discovery is the creation of *in vivo* models that recapitulating the genetic and molecular mechanisms underlying the neurodegenerative processes and that present symptoms of the HD patient. With the convergence of the genetic theory of the disease and gene transfer technology, various *in vivo* models of HD have been manufactured (Pouladi et al., 2013). Invertebrate models (*Drosophila and Caenorhabditis elegans*) have been used to effectively and rapidly screen potential therapeutic interventions and to investigate genetic/molecular pathogenesis of HD (Faber et al., 1999; Parker et al., 2001). Toxin models, which were the first animal models of HD, clarified the role of mitochondrial dysfunction and excitotoxicity, two events that have been characterized in the brains of individuals with HD (Beal et al., 1993; Pouladi et al., 2013). However, whether these models recapitulate HD biology is still uncertain (Pouladi et al., 2013). Transgenic (Tg) mouse models including R6/2, BACHD and YAC128 have been generated by introducing truncated N-terminal fragment or full-length of a juvenile HD patient’s *HTT* gene into the mouse genome (Pouladi et al., 2013; Menalled and Brunner, 2014). The Tg models revealed behavioral deficits, such as motor symptoms, electrophysiological alterations, striatal neurodegenerations, presence of intranuclear *HTT* inclusions, or alteration of transcriptional factors. However, some Tg models have various phenotypes. They do not reproduce some major features of HD pathology, such as serious neuronal degeneration in the striatum (Crook and Housman, 2011; Pouladi et al., 2013; Menalled and Brunner, 2014). R6/2 mice are well known to have limited lifespan (Perry et al., 2010). Nonetheless, these models have been widely used to study HD pathogenesis and for therapeutic trials (Crook and Housman, 2011; Pouladi et al., 2013; Menalled and Brunner, 2014). Drug discovery trials using these models are a labor-, time-, and cost-intensive efforts. Recent advances in viral-vector technology have provided promising alternatives based on direct transfer of genes to selected sub-regions of the brain (Ruiz and Déglon, 2012; Blessing and Déglon, 2016; Saraiva et al., 2016).

Recombinant adeno-associated viral (AAV) vectors have been successfully used to locally or systemically enhance or silence gene expression in a variety of tissues, including brain tissues, in adult animals or human clinical trials (de Backer et al., 2010; Ruiz and Déglon, 2012; Blessing and Déglon, 2016; Saraiva et al., 2016). The AAV vectors are reportedly more effective than lentiviral vectors at regulating the expression of specific genes at specific brain regions (de Backer et al., 2010; Ruiz and Déglon, 2012; Blessing and Déglon, 2016; Saraiva et al., 2016). Thus far, 11 serotypes of AAV have been identified. These serotypes differ in their tropism or the type of cells they infect (Kwon and Schaffer, 2008). Hybrid capsids derived from multiple different serotypes can also alter viral tropism. AAV serotype DJ (AAV-DJ), one common hybrid example, contains a hybrid capsid derived from eight serotypes. AAV-DJ displays a higher transduction efficiency *in vitro* than any wild type serotype. *In* *vivo*, it displays very high infectivity across a broad range of cell types (Grimm et al., 2008).

An AAV-induced animal model was first manufactured to express expanded polyglutamine tracts (97Q) fused to green fluorescence protein (GFP) in the adult rat brain. The model revealed the rapid formation of fibrillar, cytoplasmic and ubiquitinated nuclear polyglutamine aggregates in neurons (Senut et al., 2000). Recently, a rat HD model obtained by AAV serotype 2/9 containing Exon 1-Q138 mutant *HTT* (Q138) was reported as a short-term model for *in vivo* studies in drug discovery (Ceccarelli et al., 2016). Although these models are useful in HD studies, more models would provide more options to researchers. In the present study, we used AAV-DJ expressing N-terminal truncated fragment (N171) carrying 82 or 18 CAG repeats (mutant *HTT* and wild type, respectively) to establish and optimize a mouse model of HD. AAV-DJ-N171-82Q (AAV-DJ-82Q) and AAV-DJ-N171–18Q (AAV-DJ-18Q) as control were stereotaxically injected in the bilateral striata of juvenile mice. As a result, we demonstrate that this AAV-DJ-82Q model could be valuable as a new tool for drug efficacy trials through confirming reduced motor activity, striatal cell death and increased *HTT* aggregation.

## Materials and Methods

### AAV Vector Production

To generate AAV-*HTT*-N171-82Q, we used HD-N171-82Q cDNA construct in pBluescript to generate N171-82Q mouse model of HD (Schilling et al., 1999, 2004). The construct with first three exons (171 amino acids) of human *HTT* and 82 CAG gene repeats (*HTT*-N171-82Q) was obtained from Dr. David R. Borchelt (Santa Fe Health Care Alzheimer’s Disease Research Center, University of Florida). The *HTT*-N171-82Q construct was double digested with EcoRI/XhoI and inserted into multi-cloning sites of an AAV-MCS expression vector (Cell Biolabs Inc., cat# VPK-410). This vector was created by expression under cytomegalovirus (CMV) immediate early enhancer and promoter. As a control vector, *HTT* cDNA containing 18Q CAG repeats was used. All plasmid constructs were verified by nucleotide sequencing. These viral vectors used in this study were pseudo-typed where the transgene was flanked by inverted terminal repeats of AAV2 packaged in an AAV-DJ capsid. AAV-DJ was engineered using a DNA family shuffling technology to create a hybrid capsid from eight AAV serotypes. Additionally, AAV-DJ-*HTT*-N171-82Q and AAV-DJ-*HTT*-N171–18Q vectors were purified by iodixanol gradient ultracentrifugation by Korea Institute of Science and Technology (KIST) Virus Facility^1^. The production titer was 1.3 × 10^13^ genome copies/ml (GC/ml) for AAV-DJ-*HTT*-N171-82Q and 1.6 × 10^13^ GC/ml for AAV-DJ-*HTT*-N171–18Q.

### Animals and Ethical Statement

Male C57BL/6 mice (Narabiotec Co., Ltd., Seoul, South Korea; Seed mice were originated from Taconic Biosciences Inc., Cambridge, IN, USA) were kept at a constant temperature of 23 ± 2°C with a 12-h light-dark cycle (light on 08:00–20:00) and fed food and water *ad libitum*. The animals were allowed to habituate to the housing facilities for 1 week before the experiments. This study was carried out in accordance with the recommendations of a NIH Workshop on preclinical models of neurological diseases (Landis et al., 2012). The protocol was approved by the Institutional Animal Care and Use Committee at Kyung Hee University. In this process, proper randomization of laboratory animals and handling of data were performed in a blinded manner.

### Experimental Groups

To confirm the effect of AAV-DJ-82Q-infection, 4-week-old male juvenile mice were selected from a 5-day preliminary behavioral test. The selected mice were randomly divided into sham (*n* = 5), AAV-DJ-18Q (7.9 × 10^12^ GC/ml, *n* = 7), AAV-DJ-82Q (0.62 × 10^12^ GC/ml, *n* = 7), and AAV-DJ-82Q (1.23 × 10^12^ GC/ml, *n* = 7) groups.

### Stereotactic Brain Surgery

Mice (4 weeks; body weight, 14–15 g) were deeply anesthetized (2%–3% isoflurane, 60% O_2_, 40% N_2_O), placed on a stereotaxic apparatus (myNeuroLab, St. Louis, MO, USA), and gently fixed with ear bars and head holder. Two microliters of AAV-DJ-18Q (7.9 × 10^12^ GC/ml) and AAV-DJ-82Q (0.62 × 10^12^ GC/ml and 1.23 × 10^12^ GC/ml) were injected at a speed of 0.5 μl/min into the mid-coronal level of the bilateral striatum (stereotaxic coordinates in mm with reference to the bregma were anteroposterior, +0.8; mediolateral, ±2.0; dorsoventral, −2.5; Paxinos and Watson, 1998) using a Hamilton syringe (Sigma-Aldrich, St. Louis, MO, USA) equipped with a 30 gauge sharp-tipped needle. After 5 min, the needle was removed in three intermediate steps for 3 min to minimize the backflow. After surgical suturing, the mice were kept on a warm pad until being awakened.

### Behavioral Assessment

To investigate whether AAV-DJ-82Q injection induces neurological impairment (reduced motor coordination and imbalance), we measured body weight and accomplished pole and rota-rod performance tests (Choi et al., 2018) a weekly during 10 weeks from 1 day before AAV-DJ-82Q injection. Briefly, for the pole test, each mouse was placed on the top of a pole with a rough surface (1 cm in diameter and 50 cm in height) with its head facing upwards. The time in which the mouse completely turned downwards on the top of the pole and climbed down to the floor was recorded. At 1 h after pole test, each mouse was placed on the rotating rod (diameter = 4 cm) and tested at 16 rpm/s for 5 min. The latency to fall off the rota-rod apparatus within this time was recorded by magnetic trip plates. The assessment was accomplished by an experimenter who was unaware of the experimental condition under constant conditions of temperature (23 ± 2°C) and humidity (55 ± 5%) in a quiet room. Mice were acclimated to the pole and rota-rod apparatuses for 5 days before the first test. Mice that turned downwards on the top of the pole and climbed down to the floor within 2 min and that stayed on the rod without falling during training (for 5 min) were selected and randomly divided into the experimental groups.

### Western Blot Analysis

Western blot analysis was performed as previously described (Jang et al., 2013, 2014; Jang and Cho, 2016; Lee et al., 2016a,b). Briefly, 10 weeks (14 weeks after birth) after the AAV-DJ-82Q-injection, mice (*n* = 7 per group) were anesthetized and the striatum was immediately removed with lysis buffer. The protein (30 μg) from each striatum was transferred to polyvinylidene fluoride membranes and blocked. These membranes were probed with mouse anti-*HTT* (1:500; catalog LS-C24591-100, a.a. 181–810, clone 2Q75; LifeSpan BioSciences, Seattle, WA, USA), mouse anti-*HTT* protein (1:200; catalog MAB5374, clone EM48; Millipore, Darmstadt, Germany), rabbit anti-NeuN (1:2000; Abcam, Cambridge, MA, USA), mouse anti-dopamine- and cAMP-regulated neuronal phosphoprotein (DARPP-32) (1:2000; BD Biosciences, San Jose, CA, USA), rabbit anti-cleaved caspase-3 (1:1000; Cell Signaling Technology, Beverly, MA, USA), rabbit anti-ionized calcium-binding adapter molecule 1 (Iba-1) (1:1000; WAKO, Chuo-Ku, Japan), rabbit anti-glial fibrillary acidic protein (GFAP) (1:2000; DACO, Carpinteria, CA, USA), rabbit anti-oligodendrocyte transcription factor 2 (Olig2) (1:500; Abcam), rabbit anti-tumor necrosis factor-alpha (TNF-α) (1:500; Cell Signaling Technology), rabbit anti-interleukin (IL)-6 (1:500; Cell Signaling Technology), mouse anti-inducible nitric oxide synthases (iNOS) (1:500; Santa Cruz Biotechnology, Santa Cruz, CA, USA), mouse anti-cyclooxygenase-2 (COX-2) (1:500, BD Biosciences), rabbit anti-phospho (p)-IkappaB-alpha (IκBα) (1:500; Cell Signaling Technology), rabbit anti-p-nuclear factor kappa B (NF-κB) (1:500; Cell Signaling Technology), rabbit anti-p-signal transducer and activator of transcription 3 (STAT3) (1:500; Cell Signaling Technology), and mouse anti-GFAP (1:2000; Millipore) followed by incubation with an horseradish peroxidase-conjugated secondary antibody prior to enhanced chemiluminescence (Amersham Pharmacia Biotech, Piscataway, NJ, USA). To normalize the level of protein expression, the membranes were stripped and reprobed with mouse anti-gyceraldehyde 3-phosphate dehydrogenase (GAPDH) (1:10,000; Cell Signaling Technology). After Western blots were performed three times, the density of each band was converted into numerical values using the Photoshop CS2 program (Adobe, San Jose, CA, USA), subtracting background values from an area of film immediately adjacent to the stained band. Data are expressed as the ratio of protein to total GAPDH for each sample.

### Tissue Preparation and Immunofluorescence Procedure

To examine the histopathological changes in the striatum, 10 weeks (14 weeks after birth) after AAV-DJ-82Q injection, the mice (*n* = 7 per group) were deeply anesthetized with diethyl ether and then intracardially perfused with saline and cold 4% paraformaldehyde in 0.1 M phosphate buffer (PB, pH 7.4). Brains were removed and post-fixed for 24 h and cryoprotected in 10, 20 and 30% sucrose in PBS serially at 4°C. Serial coronal slices (30-μm thickness) of striatum were acquired on a model CM3050S freezing microtome (Leica, Wetzlar, Germany) and collected in sequence as free-floating sections on PBS. The sections from the mid-coronal level of the striatum (Franklin and Paxinos, 2008) were processed for immunofluorescence stain as previously described (Lee et al., 2016a). Briefly, the brain sections from each group (*n* = 3 per brain) were incubated with mouse anti-*HTT* protein (1:400; catalog MAB5374, clone EM48; Millipore), rabbit anti-NeuN (1:5,000; Abcam), rabbit anti-cleaved caspase-3 (1:500; Cell Signaling Technology), rabbit anti-Iba-1 (1:2,000; WAKO), mouse anti-GFAP (1:5,000; Millipore) and rabbit anti-Olig2 (1:500; Abcam) overnight with gentle agitation at room temperature. The sections were followed with a mixture of secondary antibodies and examined with confocal imaging system (LSM five PASCAL; Carl Zeiss, Germany).

### Statistical Analysis

Statistical analysis was performed by using the SPSS 21.0 package (SPSS Inc., Chicago, IL, USA) for Windows. Multiple comparisons were made using one-way ANOVA with Tukey *post hoc* test. All data are presented as means ± SEM and statistical difference was accepted at the 5% level unless otherwise indicated.

## Results

### AAV-DJ-82Q-Injection Induces Neurological Impairment

**Figure 1:**
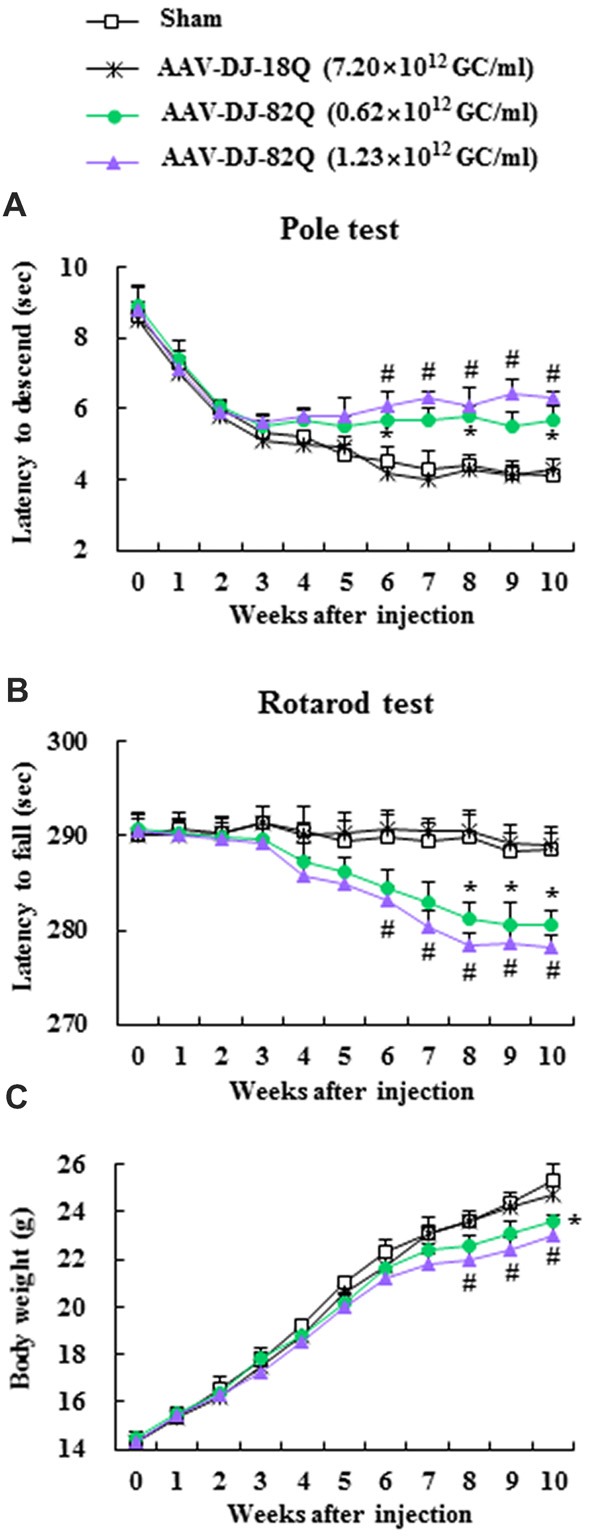
Adeno-associated viral (AAV)-DJ-82Q-injection induces neurological impairment. Two microliters of AAV-DJ-82Q (0.62 × 10^12^ GC/ml and 1.23 × 10^12^ GC/ml) and AAV-DJ-18Q (7.9 × 10^12^ GC/ml) were stereotaxically injected into the mid-striatum of male juvenile mice (4 weeks old). Sham group received the same dose of vehicle. Pole **(A)** and rota-rod performance tests **(B)** and alteration of body weight **(C)** were measured weekly. ANOVA test; *p < 0.05 and ^#^*p* < 0.05 vs. sham group and AAV-DJ-18Q groups.

First, to confirm whether AAV-DJ-82Q injection induced neurological symptoms and to select a more effective dose of the viral vector, motor coordination and balance activities were measured weekly after AAV-DJ-82Q-injetion using pole and rotarod performance tests. In the pole test, beginning at week 4 after AAV-DJ-82Q injection (8 weeks after birth), the average descent time to the bottom of the pole was increased (5.7 ± 0.3 and 6.3 ± 0.2 s in the 0.62 × 10^12^ GC/ml and 1.23 × 10^12^ GC/ml groups, respectively) 8 weeks after injection (12 weeks after birth) compared to the sham group (4.3 ± 0.5 s at 8 weeks after injection) and AAV-DJ-18Q (4.0 ± 0.3 s at 8 weeks after injection) groups (Figure 1A). In the rotarod test, the average latency to fall was reduced since week 4 after the AAV-DJ-82Q injection (282.9 ± 2.3 and 280.3 ± 1.7 s in the 0.62 × 10^12^ GC/ml and 1.23 × 10^12^ GC/ml groups, respectively, 8 weeks after injection) compared with the sham group (289.5 ± 1.9 s, 8 weeks after injection) and AAV-DJ-18Q (290.5 ± 1.3 s, 8 weeks after injection; Figure 1B). Body weight was significantly decreased since week 8 after AAV-DJ-82Q injection (1.23 × 10^12^ GC/ml), partially corresponding to both behavioral symptoms (Figure 1C). There were no significant differences in behavioral tests or change in body weight between low and high doses of vector groups (Figures 1A–C). At the end of experiment, all mice were alive. These results suggest that AAV-DJ-82Q-injection can successfully induce neurological impairment.

### AAV-DJ-82Q Injection Enhances Mutant *HTT* Expression and Induces Neurodegeneration in Striatum

**Figure 2:**
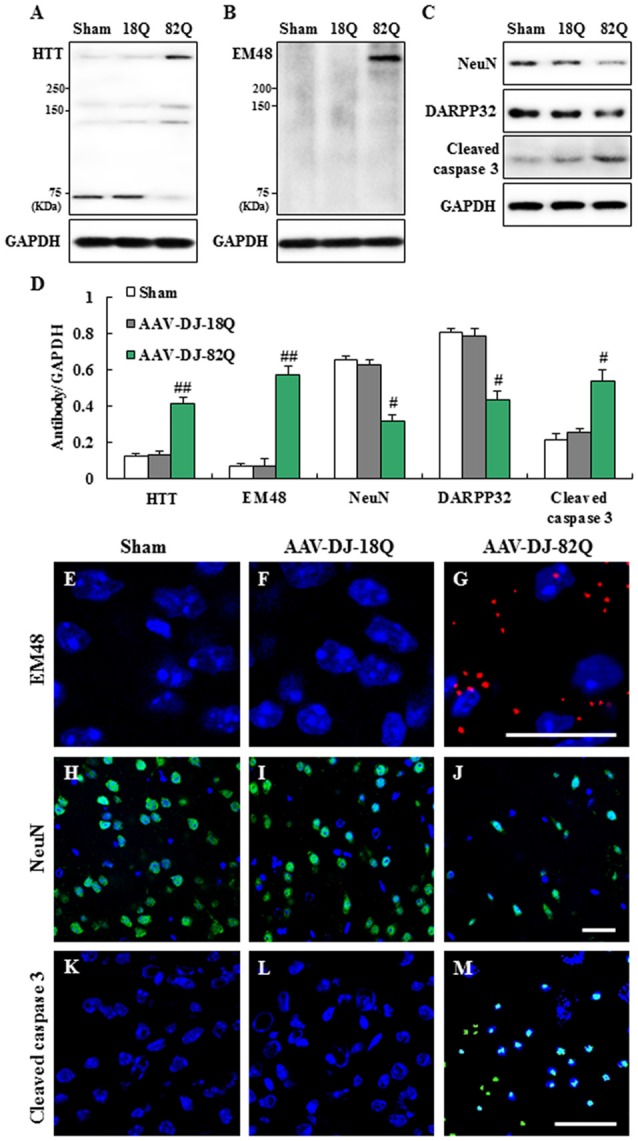
AAV-DJ-82Q injection enhances mutant *huntingtin (HTT)* expression and induces neurodegeneration in the striatum. **(A–E)** Eight weeks after AAV-DJ-82Q-injection, the striata from sham, AAV-DJ-18Q, and AAV-DJ-82Q groups (*n* = 7 per group) were analyzed by Western blot to investigate change in protein expression of *HTT* (clone, 2Q75 and EM48), NeuN, DARPP32 and cleaved caspase-3 **(A–C)** and then quantified concerning the ratio of protein to total GAPDH for each sample **(D)**. **(A–C)** show a representative Western blot. ANOVA test; ^#^*p* < 0.05 and ^##^*p* < 0.01 vs. sham and AAV-DJ-18Q groups. **(E–M)** Striatal sections (*n* = 2 per brain) from each group (*n* = 7 per group) were accomplished by immunofluorescence stain using anti-*HTT* (clone, EM48, red) **(E–G)**, NenN (green) **(H–J)**, and cleaved caspase-3 (green) **(K–M)** antibodies. Scale bar denotes 100 μm.

Since 1.23 × 10^12^ GC/ml of AAV-DJ-82Q more effectively induced neurological impairment than 0.62 × 10^12^ GC/ml of AAV, we further investigated whether the neurological impairments were associated with the level of AAV-DJ-82Q-infection into the striatum at the same dose. The level of AAV-DJ-82Q infection was measured by Western blot and immunohistochemical analysis against *HTT* and *HTT* aggregates with LS-C24591–100 and EM48 antibodies, respectively. Protein expression by anti-*HTT* antibody showed two bands. The *HTT* expression (~75 KDa) was increased clearly in the striatum of sham and AAV-DJ-18Q groups while mutant *HTT* expression was increased clearly in the striatum of AAV-DJ-82Q group (Figures 2A,D). Expression level of mutant *HTT* aggregates by using EM48 antibody was not detected in the striatum from the sham or the AAV-DJ-18Q group. However, its expression was markedly increased in the striatum of the AAV-DJ-82Q group (Figures 2B,D). In agreement with these results, many EM48-immunnoreactive cells were detected in the striatum from the AAV-DJ-82Q group, while they were not detected in the striatum from the sham and AAV-DJ-18Q groups (Figures 2E–G). Subsequently, we confirmed whether the AAV-DJ-82Q-infection induced neurodegeneration in striatum. The protein expressions of NeuN (a neuron marker) AND DARPP-32 (a marker for medium spiny neurons) were significantly reduced and cleaved caspase-3 (apoptotic cell marker) were significantly enhanced in the striatum from the AAV-DJ-82Q group compared to the sham and AAV-DJ-18Q groups (Figures 2C,D) consistent with the expression pattern of NeuN and cleaved caspase-3-immunoreactive cells (Figures 2E–M). These results suggested that AAV-DJ-82Q-injection can over-express mutant *HTT* in the striatum, resulting in neurodegeneration associated with apoptosis.

### AAV-DJ-82Q-Infection Induces Microglial and Astroglial Activation and Inflammation in Striatum

**Figure 3:**
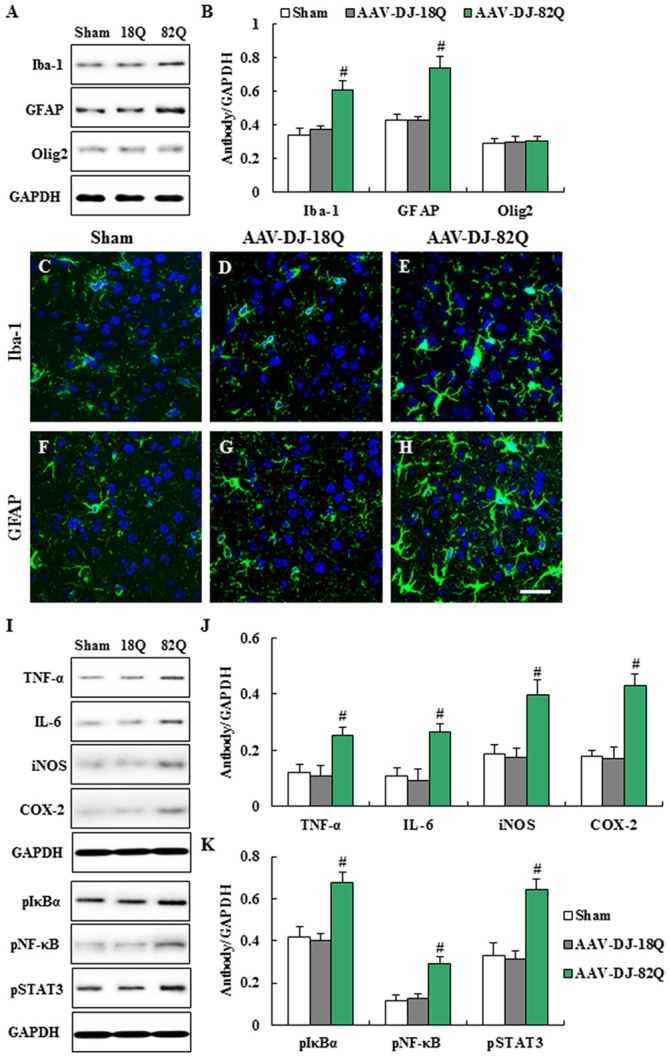
AAV-DJ-82Q-infection induces microglial and astroglial activation and 263–270 inflammation in the striatum. **(A–F)** Eight weeks after the AAV-DJ-82Q-injection, the striata from sham, AAV-DJ-18Q, and AAV-DJ-/82Q groups (*n* = 7 per group) were analyzed by Western blot to investigate change in protein expression of Iba-1, glial fibrillary acidic protein (GFAP), and Olig2 **(A)** and then quantified as the ratio of protein to total GAPDH for each sample **(B)** ANOVA test; ^#^*p* < 0.05 vs. sham and AAV-DJ-18Q groups. **(C–H)** Striatal sections (*n* = 2 per brain) from each group (*n* = 7 per group) were accomplished by immunofluorescence stain using anti-Iba-1 **(C–E)**, and GFAP **(F–H)** antibodies. Scale bar denotes 100 μm. **(I–K)** Eight weeks after AAV-DJ-82Q-injection, the striata from sham, AAV-DJ-18Q, and AAV-DJ-82Q groups (*n* = 7 per group) were analyzed by Western blot to investigate change in protein expression of TNF-α, IL-6, iNOS, COX-2, p-IκBα, p-NF-κB, p-signal transducer and activator of transcription (STAT3) and GAPDH **(I)** and then quantified concerning the ratio of protein to total GAPDH for each sample **(J,K)**. **(I)** show a representative Western blot. ANOVA test; ^#^*p* < 0.05 vs. sham and AAV-DJ-18Q groups.

We examined whether AAV-DJ-82Q infection in the striatum affects the features of neuroglia (Figure 3). The protein expressions of Iba-1 (microglia marker) and GFAP (astrocyte marker) by Western blot analysis were significantly enhanced in striatal lesions from the AAV-DJ-82Q group compared to the sham and AAV-DJ-18Q groups (Figures 3A,B). In agreement with these results, the intensity of Iba-1-immunoreactive microglia was increased within or around striatal lesions from AAV-DJ-82Q group and the immunoreactive cells displayed activated forms with an enlarged cell body and short and thick processes, as compared to the sham and AAV-DJ-82Q groups, which generally displayed the typical morphology of resting cells with small cell bodies and thin processes (Figures 3C–E), as previous described (Cho et al., 2008; Jang et al., 2013, 2014; Jang and Cho, 2016; Lee et al., 2016a). The intensity of GFAP-immunoreactive astrocytes was also increased within or around striatal lesions from the AAV-DJ-82Q group, as compared with those of the sham and AAV-DJ-18Q groups (Figures 3F–H). However, AAV-DJ-82Q-injection did not significantly affect the protein expression of Olig2 (a marker of oligodendrocytes) (Figures 3A,B). Since activated microglia and astrocytes are involved in the inflammatory response (Lobsiger and Cleveland, 2007), we measured the level of expression of representative inflammatory mediators in the striatum 8 weeks after AAV-DJ-82Q injection by Western blot analysis (Figures 3I–K). Little or no expression of TNF-α, IL-6, iNOS and COX-2 proteins were detected in the striatum of sham and AAV-DJ-18Q groups, whereas their expressions were significantly enhanced in the striatum from the AAV-DJ-82Q group (Figures 3I,J). Additionally, we measured levels of activation of NF-κB and STAT3 pathways as representative inflammatory pathways. As expected, expression levels of p-IκBα, p-NF-κB, and p-STAT3 were significantly increased in the striatum of the AAV-DJ-82Q group (Figures 3I–K). These findings suggest that the AAV-DJ-82Q-infection may produce striatal cell death and neurological dysfunction by inducing the activation of microglia, astrocytes and the inflammatory response.

### AAV-DJ-82Q Vector Infects Neurons, Microglia and Astrocytes, but Not Oligodendrocytes

**Figure 4:**
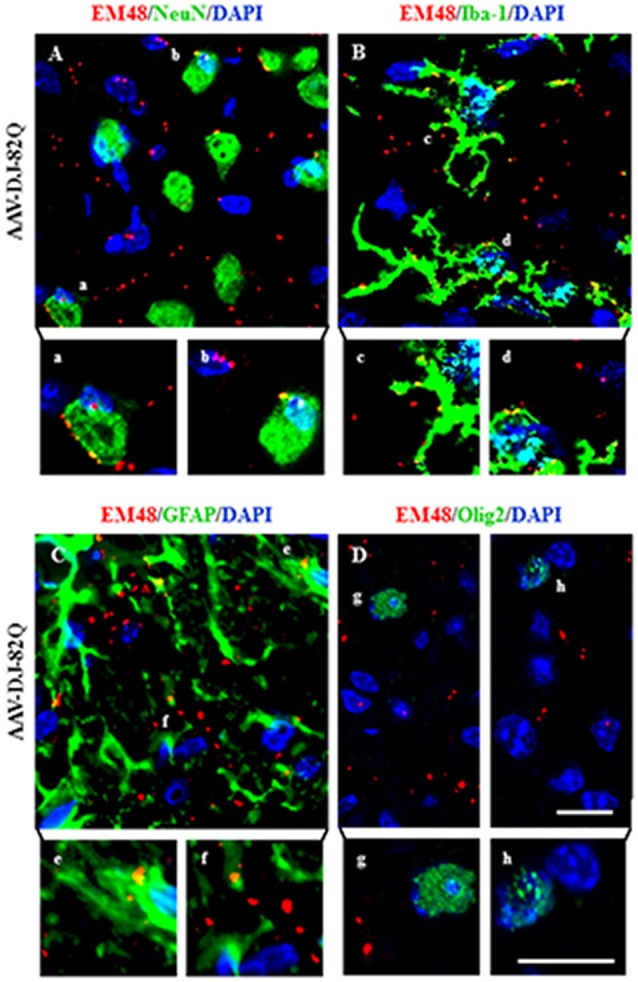
AAV-DJ-82Q vector successfully infects neurons, microglia and astrocytes. Eight weeks after the AAV-DJ-82Q-injection, the striatal sections (*n* = 2 per brain) from sham, AAV-DJ-18Q and AAV-DJ-82Q groups (*n* = 7 per group) were analyzed by double immunofluorescence stain using anti-EM48, NeuN, Iba-1, GFAP and Olig2 antibodies **(A–D)**. Lower panels in **(A–D)** display high magnification micrographs of the areas marked with **(a–h)** in upper panels. Scale bar denotes 50 μm.

The EM48-immunoreactive mutant *HTT* aggregates were densely spread in the striatal area from the AAV-DJ-82Q group (Figure 2G). However, it is unclear whether the aggregates are expressed in specific cell types. To investigate this, immunofluorescence staining was done using NeuN, Iba-1, GFAP, and Olig2 antibodies, as a marker of neurons, microglia, astrocytes, and oligodendrocytes, respectively. EM48-positive aggregates were predominantly expressed in the nucleus and cytoplasm of neurons, microglia, and astrocytes (Figures 4A–C). These aggregates were co-stained with NeuN (23.1 ± 1.5%; 45.4 ± 5.2 co-stained cells of 193.0 ± 8.7 NeuN-positive cells), Iba-1 (19.5 ± 1.3%; 14.0 ± 1.5 co-stained cells of 71.0 ± 3.6 Iba-1-positive cells), and GFAP (11.8 ± 1.1%; 8.1 ± 1.2 co-stained cells of 67.3 ± 3.9 GFAP-positive cells) antibodies (Figures 4A–C). However, these aggregates were not co-stained with Olig2 antibody (Figure 4D). These results indicate that AAV-DJ-82Q can infect specific cell types in striatum and induce HD-like pathology.

## Discussion

In the development of new therapeutic strategies for neurodegenerative disorders such as HD, animal models recapitulating the etiology, pathology and molecular mechanisms described in patients are positively necessary. However, currently a lot of animal models do not reproduce the critical therapeutic mechanisms/targets of the diseases (Crook and Housman, 2011; Pouladi et al., 2013; Menalled and Brunner, 2014). To help these challenges, various viral vectors for gene delivery and gene therapy within the nervous system have been developed over the past several decades. The studies have revealed the seemingly unlimited possibilities for viral vector systems in deciphering the nature of neurodegenerative disorders, such as HD, and so for the development of eradication regimens (Ruiz and Déglon, 2012). Despite several published reports using lentiviral and AAV vectors to model HD by over-expression of mutant *HTT*, neuroscientists in the field of HD have a much smaller selection of vectors to choose from. In the present study, we developed a novel animal model that was based on over-expression of human mutant *HTT* via a bilateral intrastriatal injection by AAV-DJ-82Q into juvenile mice. The mice over-express the transgene in the striatum beginning as juveniles and extending into adulthood. Our findings demonstrated that, after intrastriatal injection of mutant *HTT* into juvenile mice, adult mice over-expressed mutant *HTT*, displayed neuronal loss, and showed striatal neuroinflammation, resulting in motor dysfunction. In particular, mutant *HTT* predominantly infected neurons, microglia, and astrocytes. Our findings suggest that the AAV-DJ-82Q-injected mouse model may be a helpful tool to study the pathophysiology of mutant *HTT*-associated disease and to develop efficacious therapeutic strategies in translational research.

Several types of N-terminal Tg (R6/1, R6/2, N171-82Q), full-length (YAC128, BACHD) and knock-in (*Hdh*^Q111^) models have been generated to investigate the pathogenicity of HD and to discover therapeutic approaches (Pouladi et al., 2013; Menalled and Brunner, 2014). The *in vivo* models have revealed behavioral disorders including movement deficits, striatal neurodegeneration, electrophysiological dysfunctions, intracellular aggregates, and alteration of transcriptional factors (Pouladi et al., 2013; Menalled and Brunner, 2014). However, each model has different characteristics and so their results are not absolutely reproducible concerning some critical characteristics of HD pathology, such as clear neurodegeneration in the striatum or reduced life span (Crook and Housman, 2011). In addition, the research can experience difficulty in maintaining the animal strains. Problems include breeding failure and the high cost of maintaining the animals in the long-term. Our AAV-DJ-82Q model displayed clear motor deficits by pole and rota-rod tests, which were evident in the short term (at least 4–5 weeks following injection) by the onset of behavioral dysfunction as well as striatal degeneration and cellular aggregates. These results suggest that AAV-DJ-82Q model may be more helpful than others.

In the last two decades, there have been several attempts to develop the ideal vector (vehicle) for gene transfer to the central nervous system (CNS). Presently, recombinant AAVs are one of the preferred vectors, because their stable transduction of dividing and non-dividing cells, strong neural tropism, low risk of insertional mutagenesis, and reduced immune responses (Blessing and Déglon, 2016; Grieger et al., 2016; Saraiva et al., 2016). According to many studies that measured the ability of AAV serotypes to target the CNS, when administrated into the brain parenchyma of rodents, most of the serotypes (1, 2, 4, 5, 8 and 9) transduced neurons and glia in the CNS areas including striatum, hippocampus and neocortex (Ruiz and Déglon, 2012; Watakabe et al., 2015; Ceccarelli et al., 2016; Saraiva et al., 2016). Other serotypes, such as AAV-DJ, are potentially useful but have been less well examined (Cearley and Wolfe, 2006; Klein et al., 2008; Aschauer et al., 2013; Holehonnur et al., 2014). AAV-DJ is a synthetic serotype with a chimeric capsid of AAV-2, 8 and 9. AAV-DJ contains a heparin-binding domain in its capsid, which may efficiently transduce a broad range of cell types and escape from immune neutralization (Grimm et al., 2008). However, AAV-DJ has been available only recently and so relatively little is known about its optimal preparation/purification and application. In present study, we injected the striatum bilaterally with 0.62 × 10^12^ GC and 1.23 × 10^12^ GC in 2.0 ul of AAV-DJ-82Q per side, as is done with other vectors. The higher dose of viral injections produced more serious movement disorder in the pole and rota-rod performance tests (Figures 1A,B). The movement disorder progressively increased beginning 4–5 weeks following injection and lasted at least by 8 weeks after the injection. However, features of movement disorder were different to that of N171-82Q Tg mice (expressing a mutant N-terminal fragment of *HTT*), which displayed loss of coordination, hunched posture, tremors, abnormal gait, and clasping of hindlimbs (Schilling et al., 1999). In the present study, *HTT* aggregates were observed only in the striatum, similar to the *in vivo* rat HD model obtained by stereotaxic injection of AAV serotype nine containing Exon1-Q138 mutant *HTT* (AAV-9-Q138) (Ceccarelli et al., 2016). However, the expression pattern was different to that of N171-82Q Tg mice (Schilling et al., 1999). In the latter, nuclear inclusions were observed in various areas of the brain including the cerebral cortex, striatum, hippocampus, and amygdala. Furthermore, neuritic aggregates were seen in several areas of the brain including the medial amygdala and subthalamic nucleus (Schilling et al., 1999). In the present study, *HTT* aggregates were observed in the nucleus and cytoplasm of neurons, microglia and astrocytes, but not in oligodendrocytes of striatum (Figure 4). However, in three HD mouse models that express either full-length *HTT* (*Hdh*^Q150^, zQ175) or an N-terminal exon1 fragment (R6/2) of mutant HTT, the nuclear inclusions were observed in neurons, microglia, astrocytes, and oligodendrocytes (Jansen et al., 2017). In the present study, mutant* HTT* aggregates were observed in 23.1 ± 1.5% of the NeuN-positive neurons, 19.5 ± 1.3% of the Iba-1-positive microglia, and 11.8 ± 1.1% of the GFAP-positive astrocytes 8 weeks after AAV-DJ-82Q injection. However, in the three HD mouse models, at late stages of the disease, nuclear inclusions were found in 30%–50% of the neurons, 30% of the S100B-positive glial cells, 4%–10% of the GFAP-positive astrocytes, 3%–10% of the oligodendrocytes, and 0%–2% of the microglia (Jansen et al., 2017). Nuclear inclusions were also present in neurons and all studied glial cell types in human patient material (Jansen et al., 2017). Like this, in our AAV-DJ-82Q model, the expression pattern of mutant *HTT* aggregates was nearly the same with the Tg mouse models previous reported. The small differences may be explained by fundamental difference between Tg model (congenital) and AAV model (acquired) or among the AAV vector serotypes. Taken together, our results demonstrate that the AAV-DJ-82Q vector can possibly infect most functional classes of CNS, although our current data do not discriminate endothelial cell and immature/undifferentiated neural cells.

In the neurodegenerative diseases, microglia, resident immunocompetent and phagocytic cells, are activated and recruited/infiltrated around or into the lesions and serves as scavenger cell (Lobsiger and Cleveland, 2007; Kabba et al., 2018). Circulating peripheral immune cells (macrophages) may surpass a compromised blood–brain barrier and encounter neurons and microglia (Kadiu et al., 2005; Main and Minter, 2017). Although this response is initiated to protect the CNS from the harmful agents, the effect may be detrimental via releasing toxic mediators in neurodegenerative lesions (Lobsiger and Cleveland, 2007). Astrocytes can be activated by products from dead cells or infiltrated/activated immune cells within or around the lesion (Lobsiger and Cleveland, 2007). Interestingly, AAV-DJ-82Q injection clearly induced microglial and astroglial activation and protein expression of representative proinflammatory cytokines (TNF-α, IL-6) and inflammatory mediators (COX-2 and iNOS) in striatum at 14 weeks after injection, corresponding to enhanced expression of p-IκBα, p-NF-κB and p-STAT3 as representative inflammatory pathways. Whether glial activation protects against mutant *HTT* aggregates (toxicity) after the injection of AAV-DJ-82Q into normal mice is unclear. Nonetheless, our results provide the first demonstration that the AAV-DJ-82Q may induce neuroinflammatory response related to neurodegeneration in striatum.

## Conclusion

An *in vivo* model recapitulating some cardinal pathogenesis of HD needs to be generated. Here, intrastriatal injection of AAV-DJ-82Q to juvenile mice successfully induced mutant *HTT* aggregation, neurodegeneration, and neuroinflammatory response in adult striatum, resulting in HD-like symptoms. Our findings suggest that AAV-DJ-82Q vector might be a helpful tool to better understand neuropathological mechanisms in the striatum of HD patients and develop new therapeutic strategies for HD-like symptoms.

## Author Contributions

MJ performed the stereotaxic injection, behavioral experiment, immunohistochemistry and Western blots and prepared the figures. SEL developed viral vector and produced AAV virus. I-HC conceived all experiments, analyzed the results and wrote the manuscript. All authors have read and approved the final manuscript.

## Conflict of Interest Statement

The authors declare that the research was conducted in the absence of any commercial or financial relationships that could be construed as a potential conflict of interest.

## Footnotes

^1^http://virus.kist.re.kr
